# Changes in Morphometric Traits of Ground Beetles Along Urbanization Gradients

**DOI:** 10.1093/jisesa/iez127

**Published:** 2020-01-11

**Authors:** Dalma Papp, Szabolcs Mizser, Leila Nagy, Andreas Vidic, Edina Simon, Béla Tóthmérész

**Affiliations:** 1 Department of Ecology, University of Debrecen, Egyetem tér 1, Debrecen H-4032, Hungary; 2 MTA-DE Biodiversity and Ecosystem Services Research Group, Egyetem tér 1, Debrecen H-4032, Hungary; 3 Department für Naturschutzbiologie, Vegetations- und Landschaftsökologie, Universität Wien, Althanstrasse 14, 1090 Wien, Austria

**Keywords:** Carabidae, bioindication, asymmetry, morphological abnormality, urban stress gradient

## Abstract

Urbanization has a significant impact on abiotic and biotic factors in nature. We examined the morphometric characters of four carabid species (*Abax parallelepipedus*, *Carabus scheidleri*, *Carabus violaceus*, and *Pterostichus oblongopunctatus*) along urbanization gradients in and around the cities of Vienna (Austria) and Debrecen (Hungary). We found significant differences among urban, suburban, and rural areas in the parameters of antennomers, the maxillary palpus, the labial palpus, and the length of the tibia and the elytra of the carabids studied. We also found significant differences between males and females based on the parameters of antennomers, the maxillary palpus, the labial palpus, the femur, and the elytra. An interaction between urbanization and sex was found in the case of antennomers, the maxillary palpus, the labial palpus, the femur, and the elytra. Our findings suggested that in the cases of species from Carabini tribus the parameters of antennomers, the maxillary palpus, and the elytra could be useful for assessing the effects of urbanization because these morphometric characters responded sensitively to the environmental stress, whereas the most useful parameters are those of antennomers and the tibia for the species of Pterostichini tribus. Our findings also revealed that females are more sensitive to environmental stress than males.

The effects of human activities have a significant impact on terrestrial ecosystems. A wide variety of emitted substances (mostly trace elements) directly and indirectly influences abiotic and biotic factors in urbanized areas (Gál et al. 2008). The emitted chemical and biological pollutants can change environmental conditions and thereby affect the biodiversity and also the conditions of organisms (Clarke 1993, Peters et al. 2001, Cachada et al. 2013). In addition, unnatural climatic conditions, increasing traffic density and buildup rates, habitat fragmentation, and isolation can also result in specific changes in habitat quality and bring environmental stress (Saunders et al. 1991, Ikeda 2006). Due to the continuously changing environmental factors, the diversity and community structure of invertebrates may change significantly in urban habitats compared with rural ones (Faeth et al. 2005, Biaggin et al. 2007, McKinney 2008, Lagucki et al. 2017). In extreme cases, populations become extinct from areas, which are affected by anthropogenic stress (Acevedo-Whitehouse and Duffus 2009). However, the damage can also be detected at moderate levels such as in abnormalities of their life cycle and reproduction, as well as in morphometric changes whose effects have been less widely investigated in previous studies (Peakall 1992, Eeva et al. 2000). Changes in the metric parameters of arthropods can be successfully used as indicators of anthropogenic disturbance (Lagisz 2008). In bioindicator arthropods, contaminations and habitat degradation have a significant effect on the occurrence of morphological abnormalities (Cattaneo et al. 2004). Abiotic factors such as nutrition quality, buildup rate, and habitat fragmentation can also vary greatly among habitats and affect the development and morphometry of these organisms (Parsons 1992, Weller and Ganzhorn 2003, Vandewoestijne et al. 2005, Shingleton et al. 2007).

Arthropod species occur in various habitats. Due to their life cycle, high density, and variability, they are widely used as indicators in environmental risk assessments (Zöld and Wittmann 2003, Magura et al. 2010). Stress factors can cause morphological abnormalities such as lower body mass and/or asymmetry of the body. To survive, individuals living in disturbed habitats concentrate more energy on elimination processes (Cortet et al. 1999). Thus, a lower amount of energy is used for developmental processes. Under constant conditions the development of the phenotype is stable, but overcoming stress factors can induce morphological disorders such as decreased symmetry and body size (Van Dongen et al. 2001, Lens et al. 2002). Stress-induced asymmetry and the relationship between asymmetry and urbanization were also tested for a variety of arthropod species (Godet et al. 2012, Olivero et al. 2014). In some cases, the morphological abnormalities were associated with air, soil, and water pollution; pesticides; poor nutrition quality; food deficiency; and isolating factors such as artificial barriers (Weller and Ganzhorn 2003, De Anna et al. 2013).

Ground beetles (Coleoptera: Carabidae) are well known both taxonomically and ecologically. These species are sensitive indicators of biotic and abiotic pressure; thus, they are frequently used to explore the effects of soil pollution, forest management, and tourist flow (Venn 2007, Sukhodolskaya 2013, Tőzsér et al. 2019). Changes in morphological parameters such as the body size and symmetry of carabid beetles potentially indicate different types of extrinsic stress factors (McGeoch 1998). There is a highly significant relationship between the functional morphological trait characters of carabid species and the characteristics of their habitat (Ribera et al. 2001, Weller and Ganzhorn 2003). Thus, carabid species are useful for the investigation of anthropogenic effects. Although many studies have investigated the impacts of urbanization on beetle diversity and richness, fewer studies have been performed regarding the responses of species’ morphometric traits (e.g., body size and asymmetry). Because reduced body size and the disruption of symmetry reflect severe environmental stresses and/or inbreeding depression, which can be a sign of population decline and local extinction, these need to be explored in the context of urbanization.

The aim of our study was to examine morphological traits of four ground beetle species (*Abax parallelepipedus*, *Carabus scheidleri*, *Carabus violaceus*, and *Pterostichus oblongopunctatus*) along an urbanization gradient in and around the cities of Vienna and Debrecen. The following metric traits were measured in all studied species, based on the earlier study by Elek et al. (2014): the lengths of three antennomers of the right and left antennae, the lengths of the femur and tibia of the front legs, and the lengths of the right and left elytra (inner edges). Two palpomers of the right and left maxillary palpus and labial palpus were measured in *A. parallelepipedus*, *C. scheidleri*, and *C. violaceus*. We hypothesized that the tested morphometric traits change under different levels of anthropogenic stress. Therefore, we predicted that 1) along an urbanization gradient detectable differences are found based on Carabid morphometry, 2) the size of the measured traits is greater in males than in females, and 3) the symmetry of morphological traits is affected by urbanization; thus, the tested areas could be separated from each other based on the level of asymmetry of the beetles’ bodies.

## Materials and Methods

### Study Area and Sampling Design

Sampling areas were located in and around the cities of Vienna (Austria) and Debrecen (Hungary) similarly to the globenet protocol (Niemelä et al. 2000). We used an identical sampling design in both countries; based on Simon et al. (2013, 2016), a gradient was chosen to represent the effects of urbanization on morphological characteristics in various habitats. Along the gradient three areas were selected with increasing levels of urbanization: rural, suburban, and urban. Four spatial replicates (sites) were selected within each sampling area. In each site, 20 traps were used. Thus, in summary, 80 traps were used in each area. Adult carabids were collected by trapping during their reproductive period from May to August in 2010 in both cities. Until the measurement processes, the samples were stored in polyethylene bags at −18°C.

There were *A. parallelepipedus* (Pterostichini tribus) and *C. scheidleri* (Carabini tribus) specimens collected in and around the city of Vienna along an urbanization gradient. From the studied areas, the number of *A. parallelepipedus* specimens was 47 (urban: *n* = 10, suburban: *n* = 20, rural: *n* = 17), and the number of collected *C. scheidleri* specimens was 55 (urban: *n* = 18, suburban: *n* = 19, rural: *n* = 18).

In and around Vienna, all sampling areas of the gradient were characterized by high habitat dynamics, and specific soil parameters and vegetation types. The urban area was located in the Wiener Prater (48°12′N, 16°24′E) near the city center in a forested park. In this area, the proportion of green space was 39.1%, and the transport system is remarkable in this area of the city. The suburban area was in the district of Leopoldstadt (48°11′N, 16°26′E) where the green space made up 63.5% of the total area. The rural area (48°10′N, 16°31′E) was located within the Donau-Auen National Park along the Donau-Oder-Kanal. In this area, the green space was 99.5%, and forest management, traffic, and tourism intensity were reduced.

There were *C. violaceus* (Carabini tribus) and *P. oblongopunctatus* (Pterostichini tribus) specimens collected in and around Debrecen along an urbanization gradient similar to that in Vienna. The number of *C. violaceus* was 188 (urban: *n* = 98, suburban: *n* = 13, rural: *n* = 77), and the number of *P. oblongopunctatus* was 57 (urban: *n* = 25, suburban: *n* = 11, rural: *n* = 21). The urban area was located within a forested park in the city center (47°32′N, 21°37′E) similar to the studied urban area in Vienna. This area was characterized by a high traffic load. The suburban area (47°33′N, 21°38′E) was located between urban and rural areas. These forest fragments are characterized by moderate anthropogenic disturbance, and there was park maintenance activity. The rural area (47°34′N, 21°37′E) was located in a forested area outside Debrecen (Simon et al. 2014).

### Morphometry of Carabid Beetles

**Fig. 1.**
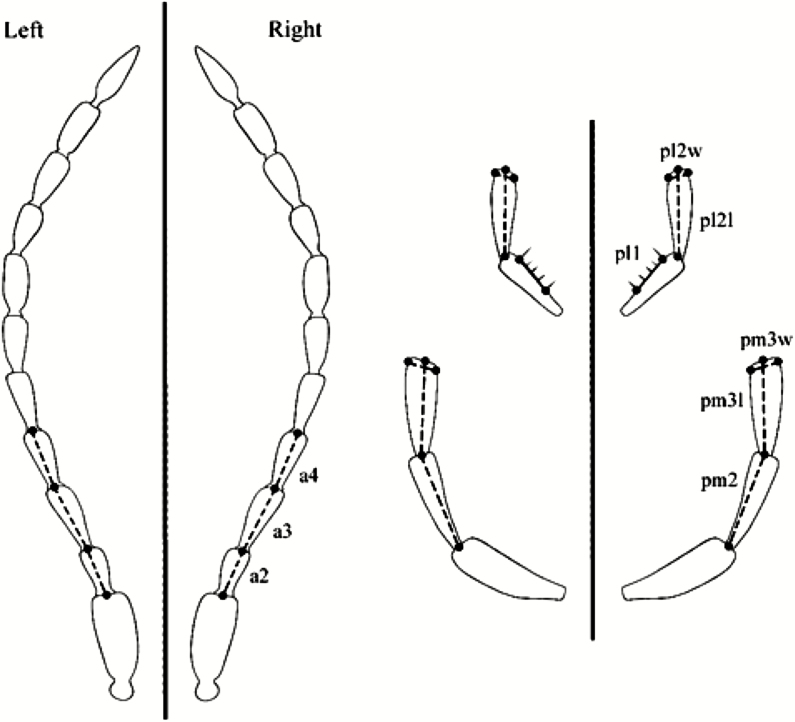
The measured morphological traits. Notations: a2, length of the second segment (antennomer) of the antenna; a3, length of the third segment (antennomer) of the antenna; a4, length of the fourth segment (antennomer) of the antenna; pm2, length of the second palpomer of the maxillary palpus; pm3l, length of the third palpomer of the maxillary palpus; pm3w, width of the third palpomer of the maxillary palpus; pl1, distance between the two extreme setae on the first palpomer of the labial palpus; pl2l, length of the second palpomer of the labial palpus; pl2w, width of the second palpomer of the labial palpus.

**Fig. 2.**
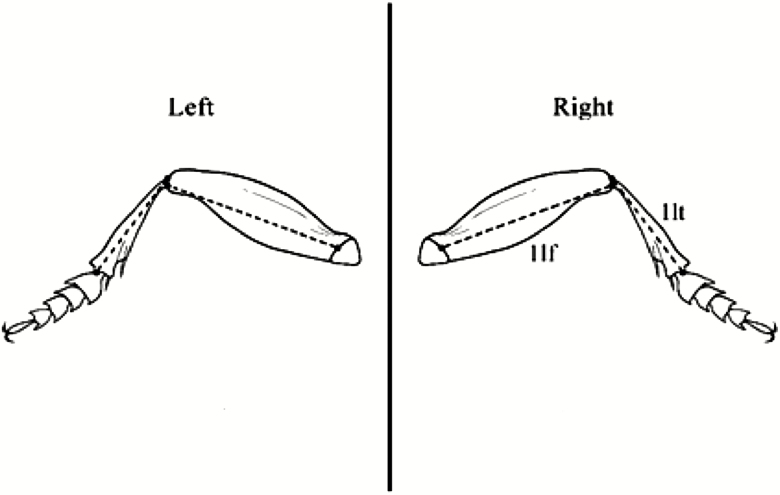
The measured morphological traits. Notations: 1lf, femur length of the front leg, 1lt, length of tibia of the front leg.

**Fig. 3.**
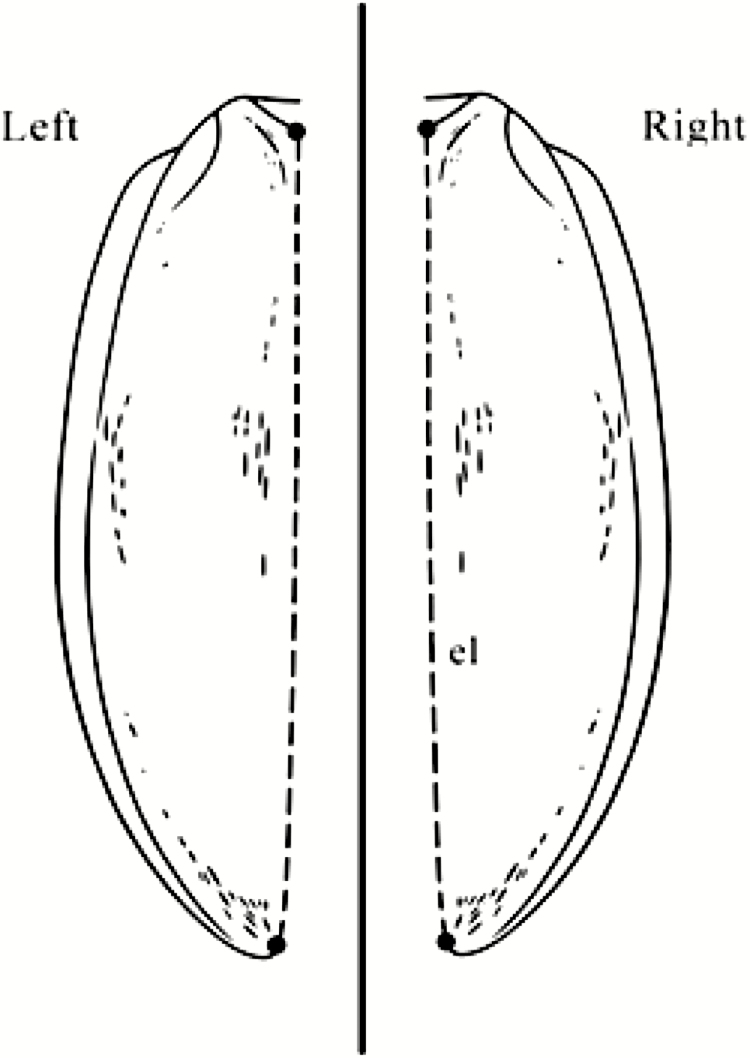
The measured morphological traits. Notation: el, length of the elytron (inner edges).

During data acquisition, the following metric traits were measured in all species studied, based on the earlier study by Elek et al. (2014): 1) the lengths of the right and left second, 2) third, and 3) fourth segments of the antennomers, 4) the lengths of the first femora, 5) the first tibiae, and 6) the lengths of the elytra (inner edges; Figs. 1–3). 7) The lengths of the second palpomer and 8) the lengths and 9) widths of the third palpomer of the right and left maxillary palpus; 10) the distance between the two extreme setae on the first palpomer; and 11) the widths and 12) lengths of the second palpomer of the right and left labial palpus were measured in *A. parallelepipedus*, *C. scheidleri*, and *C. violaceus* (Fig. 1). The removal process of maxillary palpus and labial palpus was impracticable for *P. oblongopunctatus* due to the small average body size (10.3 mm) of this species (Hůrka 1996). Each individual was measured three times. A digital camera (Olympus C-7070) attached to a stereomicroscope (Olympus SZX7) was used to photograph the ground beetles. During the photo documentation, the samples were kept wet; thus, the original lengths and shapes of individuals were maintained. The measurement of the metric traits was performed by the WinImag 1.0 data acquisition system.

### Data Analysis

SPSS/PC+ and CANOCO program packages were used during the statistical analyses. The homogeneity of variances was tested by Levene’s test. Principal component analysis (PCA) was used to explore the differences in the measured traits. Generalized linear model (GLM) was used to test the differences in the morphological traits of the studied species in the studied areas (urban, suburban, and rural), and the sex of the specimens (female and male), and for the interaction analysis between urbanization and sex. Asymmetry (right and left) was tested by the Mann–Whitney *U* test.

## Results

**Fig. 4.**
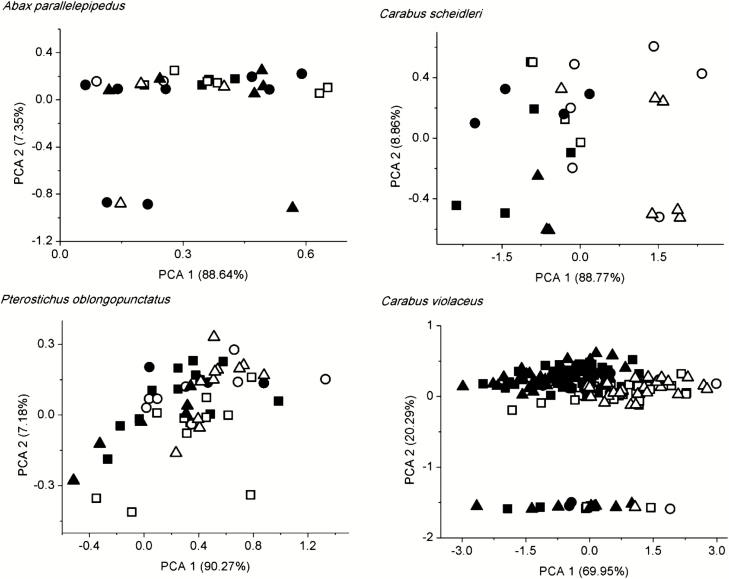
Principal component biplot of morphological traits of the studied carabid species. Notations: filled square—males from urban areas, blank square—females from urban areas, filled circle—males from suburban areas, blank circle—females from suburban areas, filled triangle—males from rural areas, blank triangle—females from rural areas.

Using PCA, there were overlaps among the studied areas (urban, suburban, and rural) based on morphological traits (Fig. 4). In the case of sex, there was little overlap between males and females based on the morphological traits by PCA (Fig. 4).

Using GLM, a significant difference was found among areas in the width of the second palpomer of the labial palpus and the length of the tibia in the case of *A. parallelepipedus*. The greatest width of the second palpomer of the labial palpus was found in the suburban, whereas the greatest length of the tibia was found in the urban area. In the case of *C. scheidleri*, the greatest length of the second segment of the antenna and the greatest width of the third palpomer of the maxillary palpus were found in the urban area. The length of the third segment of the antenna, the length of the fourth segment of the antenna, and the width of the second palpomer of the labial palpus were all greater in the surburban area than in the rural area. Based on the measured traits of *C. violaceus*, the greatest length of the fourth segment of the antenna, and the greatest length and width of the third palpomer of the maxillary palpus, and the greatest length of the elytra were found in the suburban area. In the case of *P. oblongopunctatus*, the length of the third segment of the antenna was the highest in the rural area, whereas the length of the fourth segment of the antenna and the length of tibia was the highest in the suburban area (Tables 1–3).

**Table 1.** Descriptive statistics of morphological traits (mean ± SE) of *Carabus scheidleri* and *Abax parallelepipedus*

	Traits	Urban				Suburban				Rural			
		Male		Female		Male		Female		Male		Female	
		Right	Left	Right	Left	Right	Left	Right	Left	Right	Left	Right	Left
*Carabus scheidleri*	a2	0.93 + 0.02	0.93 + 0.02	0.92 + 0.07	0.90 + 0.07	0.93 + 0.03	0.95 + 0.03	0.89 + 0.01	0.89 + 0.02	0.78 + 0.03	0.83 + 0.06	0.92 + 0.02	0.94 + 0.02
	a3	1.57 + 0.03	1.56 + 0.03	1.39 + 0.10	1.42 + 0.10	1.58 + 0.04	1.53 + 0.05	1.49 + 0.02	1.48 + 0.02	1.35 + 0.06	1.35 + 0.05	1.57 + 0.03	1.55 + 0.3
	a4	1.32 + 0.02	1.31 + 0.03	1.29 + 0.09	1.26 + 0.09	1.31 + 0.03	1.30 + 0.03	1.23 + 0.0	1.22 + 0.02	1.14 + 0.03	1.15 + 0.03	1.32 + 0.03	1.29 + 0.02
	pm2	1.03 + 0.02	1.03 + 0.02	1.02 + 0.02	1.02 + 0.03	1.08 + 0.03	1.11 + 0.05	1.08 + 0.03	1.09 + 0.03	1.01 + 0.02	0.99 + 0.03	1.03 + 0.02	1.03 + 0.02
	pm3l	1.10 + 0.02	1.07 + 0.03	1.09 + 0.05	1.09 + 0.04	1.14 + 0.03	1.14 + 0.03	1.09 + 0.04	1.11 + 0.03	1.11 + 0.05	1.13 + 0.05	1.06 + 0.06	1.05 + 0.06
	pm3w	0.53 + 0.01	0.52 + 0.01	0.50 + 0.03	0.50 + 0.01	0.55 + 0.02	0.54 + 0.01	0.53 + 0.01	0.54 + 0.02	0.49 + 0.02	0.50 + 0.01	0.53 + 0.01	0.53 + 0.02
	pl1	0.42 + 0.02	0.40 + 0.01	0.50 + 0.02	0.49 + 0.02	0.42 + 0.04	0.40 + 0.04	0.43 + 0.01	0.47 + 0.02	0.45 + 0.04	0.43 + 0.04	0.44 + 0.02	0.39 + 0.02
	pl2l	1.27 + 0.03	1.28 + 0.03	1.36 + 0.04	1.34 + 0.06	1.31 + 0.02	1.35 + 0.02	1.34 + 0.04	1.35 + 0.03	1.31 + 0.03	1.30 + 0.03	1.28 + 0.03	1.29 + 0.03
	pl2w	0.54 + 0.01	0.55 + 0.01	0.57 + 0.02	0.56 + 0.02	0.57 + 0.01	0.56 + 0.01	0.57 + 0.01	0.57 + 0.01	0.52 + 0.01	0.53 + 0.01	0.54 + 0.02	0.54 + 0.02
	femur	4.75 + 0.09	4.85 + 0.07	5.13 + 0.18	5.16 + 0.18	4.90 + 0.16	4.97 + 0.16	4.98 + 0.08	5.21 + 0.10	4.62 + 0.25	4.65 + 0.22	5.06 + 0.14	5.20 + 0.15
	tibia	4.82 + 0.06	4.74 + 0.05	5.21 + 0.21	5.29 + 0.16	4.82 + 0.20	4.80 + 0.17	5.12 + 0.11	5.17 + 0.14	4.60 + 0.25	4.60 + 0.23	5.08 + 0.16	5.12 + 0.15
	elytra	15.4 + 0.4	15.5 + 0.4	16.0 + 0.2	16.1 + 0.3	15.7 + 0.5	15.8 + 0.5	17.4 + 0.4	17.4 + 0.5	15.8 + 0.1	15.9 + 0.1	17.8 + 0.3	17.9 + 0.3
*Abax parallelepipedus*	a2	0.65 + 0.03	0.64 + 0.02	0.66 + 0.04	0.62 + 0.04	0.63 + 0.04	0.62 + 0.03	0.65 + 0.01	0.63 + 0.06	0.65 + 0.04	0.63 + 0.04	0.60 + 0.05	0.60 + 0.05
	a3	0.92 + 0.01	0.93 + 0.04	0.89 + 0.03	0.90 + 0.04	0.91 + 0.04	0.90 + 0.05	0.87 + 0.01	0.86 + 0.03	0.93 + 0.03	0.93 + 0.03	0.86 + 0.06	0.87 + 0.05
	a4	0.93 + 0.03	0.93 + 0.03	0.87 + 0.03	0.89 + 0.03	0.93 + 0.03	0.91 + 0.04	0.87 + 0.05	0.87 + 0.01	0.94 + 0.03	0.94 + 0.04	0.84 + 0.05	0.86 + 0.04
	pm2	0.66 + 0.03	0.67 + 0.02	0.67 + 0.03	0.67 + 0.02	0.67 + 0.04	0.68 + 0.03	0.63 + 0.03	0.65 + 0.04	0.66 + 0.02	0.66 + 0.03	0.68 + 0.02	0.67 + 0.04
	pm3l	0.78 + 0.03	0.76 + 0.01	0.76 + 0.03	0.74 + 0.05	0.73 + 0.03	0.72 + 0.04	0.75 + 0.03	0.74 + 0.04	0.75 + 0.05	0.72 + 0.05	0.73 + 0.02	0.74 + 0.02
	pm3w	0.23 + 0.01	0.24 + 0.01	0.23 + 0.01	0.24 + 0.02	0.23 + 0.01	0.24 + 0.01	0.22 + 0.02	0.24 + 0.01	0.23 + 0.01	0.24 + 0.01	0.22 + 0.01	0.23 + 0.01
	pl1	0.15 + 0.01	0.14 + 0.04	0.15 + 0.02	0.14 + 0.02	0.15 + 0.01	0.16 + 0.02	0.16 + 0.01	0.17 + 0.01	0.15 + 0.01	0.14 + 0.02	0.14 + 0.02	0.13 + 0.02
	pl2l	0.86 + 0.04	0.86 + 0.02	0.87 + 0.04	0.86 + 0.04	0.84 + 0.02	0.84 + 0.05	0.89 + 0.04	0.90 + 0.03	0.88 + 0.04	0.88 + 0.04	0.87 + 0.02	0.88 + 0.03
	pl2w	0.17 + 0.01	0.17 + 0.01	0.16 + 0.02	0.15 + 0.02	0.20 + 0.02	0.20 + 0.02	0.15 + 0.03	0.15 + 0.03	0.14 + 0.01	0.14 + 0.01	0.13 + 0.01	0.12 + 0.01
	femur	3.62 + 0.10	3.69 + 0.07	3.67 + 0.14	3.72 + 0.09	3.74 + 0.10	3.82 + 0.12	3.58 + 0.09	3.70 + 0.21	3.73 + 0.13	3.77 + 0.15	3.58 + 0.09	3.68 + 0.15
	tibia	3.67 + 0.05	3.71 + 0.02	3.69 + 0.17	3.73 + 0.15	3.61 + 0.11	3.65 + 0.16	3.51 + 0.08	3.55 + 0.09	3.67 + 0.15	3.69 + 0.13	3.58 + 0.09	3.62 + 0.06
	elytra	10.6 + 0.2	10.8 + 0.4	10.7 + 0.3	10.7 + 0.4	10.7 + 0.4	10.8 + 0.5	10.8 + 0.1	10.8 + 0.1	10.6 + 0.4	11.1 + 0.6	10.3 + 0.3	10.3 + 0.6

Notations: a2, length of the second segment of the antennomer flagellum; a3, length of the third segment of the antennomer; a4, length of the fourth segment of the antennomer; pm2, length of the second palpomer of the maxillary palpus; pm3l, length of the third palpomer of the maxillary palpus; pm3w, width of the third palpomer of the maxillary palpus; pl1, distance between the two extreme setae on the first palpomer of labial palpus; pl2l, length of the second palpomer of labial palpus; pl2w, width of the second palpomer of labial palpus; femur, length of femur in front leg; tibia, length of tibia in front leg; elytra, length of elytra, mm.

**Table 2.** Descriptive statistics of morphological traits (mean ± SE) of *Carabus violaceus* and *Pterostichus oblongopunctatus*

	Traits	Urban				Suburban				Rural			
		Male		Female		Male		Female		Male		Female	
		Right	Left	Right	Left	Right	Left	Right	Left	Right	Left	Right	Left
*Carabus violaceus*	a2	1.07 ± 0.10	1.07 ± 0.10	1.07 ± 0.10	1.09 ± 0.10	1.16 ± 0.15	1.17 ± 0.14	1.05 ± 0.10	1.06 ± 0.07	1.06 ± 0.09	1.07 ± 0.09	1.07 ± 0.06	1.10 ± 0.06
	a3	1.50 ± 0.14	1.50 ± 0.14	1.52 ± 0.13	1.52 ± 0.13	1.64 ± 0.21	1.67 ± 0.21	1.49 ± 0.09	1.46 ± 0.09	1.51 ± 0.12	1.50 ± 0.12	1.51 ± 0.09	1.51 ± 0.09
	a4	1.18 ± 0.10	1.18 ± 0.10	1.20 ± 0.10	1.20 ± 0.10	1.31 ± 0.17	1.31 ± 0.17	1.18 ± 0.09	1.20 ± 0.09	1.18 ± 0.11	1.18 ± 0.11	1.20 ± 0.08	1.20 ± 0.08
	pm2	1.07 ± 0.06	1.07 ± 0.06	1.07 ± 0.06	1.08 ± 0.07	1.11 ± 0.05	1.12 ± 0.05	1.09 ± 0.08	1.10 ± 0.08	1.09 ± 0.08	1.10 ± 0.08	1.09 ± 0.07	1.09 ± 0.07
	pm3l	1.17 ± 0.07	1.17 ± 0.07	1.14 ± 0.06	1.13 ± 0.06	1.22 ± 0.06	1.22 ± 0.05	1.20 ± 0.08	1.19 ± 0.07	1.20 ± 0.08	1.19 ± 0.07	1.17 ± 0.07	1.15 ± 0.04
	pm3w	0.77 ± 0.05	0.77 ± 0.05	0.64 ± 0.05	0.65 ± 0.05	0.78 ± 0.05	0.77 ± 0.05	0.76 ± 0.07	0.77 ± 0.06	0.76 ± 0.07	0.77 ± 0.06	0.63 ± 0.04	0.64 ± 0.06
	pl1	0.84 ± 0.17	0.89 ± 0.18	0.76 ± 0.15	0.79 ± 0.14	0.80 ± 0.15	0.86 ± 0.15	0.89 ± 0.07	0.88 ± 0.07	0.89 ± 0.18	0.88 ± 0.18	0.83 ± 0.16	0.79 ± 0.16
	pl2l	1.36 ± 0.09	1.37 ± 0.09	1.24 ± 0.08	1.26 ± 0.07	1.35 ± 0.04	1.36 ± 0.02	1.29 ± 0.06	1.31 ± 0.06	1.36 ± 0.09	1.36 ± 0.09	1.28 ± 0.09	1.30 ± 0.07
	pl2w	0.86 ± 0.06	0.87 ± 0.05	0.71 ± 0.06	0.70 ± 0.06	0.83 ± 0.05	0.84 ± 0.04	0.77 ± 0.02	0.74 ± 0.04	0.86 ± 0.06	0.87 ± 0.06	0.68 ± 0.04	0.69 ± 0.05
	femur	4.96 ± 0.29	5.03 ± 0.27	4.94 ± 0.22	5.01 ± 0.22	5.09 ± 0.25	5.17 ± 0.27	4.86 ± 0.41	5.00 ± 0.27	4.98 ± 0.26	5.01 ± 0.26	4.98 ± 0.29	5.05 ± 0.27
	tibia	4.78 ± 0.24	4.81 ± 0.24	4.84 ± 0.20	4.95 ± 0.27	4.88 ± 0.22	4.94 ± 0.21	4.76 ± 0.29	4.85 ± 0.25	4.77 ± 0.26	4.78 ± 0.28	4.90 ± 0.26	4.97 ± 0.27
	elytra	18.7 ± 0.8	18.7 ± 0.7	19.9 ± 0.9	19.9 ± 0.9	19.1 ± 0.6	19.0 ± 0.6	20.8 ± 1.2	20.8 ± 1.3	18.5 ± 1.0	18.5 ± 1.0	20.5 ± 0.7	20.4 ± 0.7
*Pterostichus oblongopunctatus*	a2	0.33 ± 0.02	0.34 ± 0.01	0.31 ± 0.02	0.33 ± 0.03	0.330 ± 0.02	0.34 ± 0.04	0.34 ± 0.02	0.35 ± 0.03	0.34 ± 0.02	0.33 ± 0.02	0.33 ± 0.03	0.33 ± 0.04
	a3	0.54 ± 0.03	0.54 ± 0.04	0.53 ± 0.03	0.52 ± 0.03	0.55 ± 0.01	0.54 ± 0.01	0.55 ± 0.03	0.54 ± 0.03	0.57 ± 0.03	0.57 ± 0.03	0.55 ± 0.04	0.56 ± 0.03
	a4	0.49 ± 0.04	0.50 ± 0.03	0.48 ± 0.03	0.48 ± 0.03	0.51 ± 0.02	0.50 ± 0.03	0.51 ± 0.03	0.51 ± 0.03	0.52 ± 0.03	0.52 ± 0.02	0.49 ± 0.03	0.50 ± 0.03
	femur	2.09 ± 0.11	2.06 ± 0.08	2.02 ± 0.15	2.01 ± 0.15	2.15 ± 0.05	2.11 ± 0.06	2.12 ± 0.06	2.09 ± 0.08	2.01 ± 0.11	2.01 ± 0.11	2.13 ± 0.11	2.10 ± 0.11
	tibia	2.24 ± 0.09	2.18 ± 0.10	2.12 ± 0.14	2.04 ± 0.17	2.28 ± 0.05	2.26 ± 0.02	2.23 ± 0.09	2.18 ± 0.08	2.16 ± 0.13	2.11 ± 0.09	2.22 ± 0.10	2.18 ± 0.12
	elytra	6.47 ± 0.30	6.49 ± 0.46	6.50 ± 0.36	6.64 ± 0.39	6.53 ± 0.51	6.79 ± 0.46	6.64 ± 0.47	6.64 ± 0.44	6.22 ± 0.32	6.26 ± 0.37	6.68 ± 0.22	6.71 ± 0.22

Notations: a2, length of the second segment of the antennomer flagellum; a3, length of the third segment of the antennomer; a4, length of the fourth segment of the antennomer; pm2, the length of the second palpomer of the maxillary palpus; pm3l, length of the third palpomer of the maxillary palpus; pm3w, width of the third palpomer of the maxillary palpus; pl1, distance between the two extreme setae on the first palpomer of labial palpus; pl2l, length of the second palpomer of the labial palpus; pl2w, width of the second palpomer of the labial palpus; femur, length of the femur in front leg; tibia, length of the tibia in front leg; elytra, length of the elytra, mm.

**Table 3.** *P* values of morphological traits based on carabid species, studied areas, sex, and interaction between areas and sex

	*Abax parallelepipedus*			*Carabus scheidleri*			*Carabus violaceus*			*Pterostichus oblongopunctatus*		
	Area	Sex	Area × sex	Area	Sex	Area × sex	Area	Sex	Area × sex	Area	Sex	Area × sex
a2	0.343	0.218	0.059	0.008	0.161	<0.001	0.225	0.055	0.015	0.309	0.623	0.314
a3	0.208	0.002	0.341	0.023	0.806	<0.001	0.179	0.017	<0.001	0.001	0.201	0.745
a4	0.478	<0.001	0.050	0.013	0.369	<0.001	0.036	0.123	0.009	0.015	0.123	0.110
pm2	0.919	0.449	0.658	<0.001	0.559	0.518	0.012	0.648	0.953	n.m.	n.m.	n.m.
pm3l	0.233	0.590	0.085	0.216	0.138	0.233	0.003	<0.001	0.891	n.m.	n.m.	n.m.
pm3w	0.569	0.129	0.767	<0.001	0.952	0.012	0.113	<0.001	0.340	n.m.	n.m.	n.m.
pl1	0.017	0.916	0.602	0.387	0.021	0.012	0.359	0.022	0.229	n.m.	n.m.	n.m.
pl2l	0.482	0.101	0.086	0.196	0.204	0.169	0.116	<0.001	0.028	n.m.	n.m.	n.m.
pl2w	<0.001	<0.001	<0.001	0.002	0.053	0.866	0.097	<0.001	0.004	n.m.	n.m.	n.m.
femur	0.763	0.027	0.056	0.305	<0.001	0.139	0.605	0.119	0.200	0.049	0.855	0.001
tibia	0.023	0.139	0.325	0.245	<0.001	0.545	0.957	0.203	0.073	0.008	0.069	<0.001
elytra	0.233	0.148	0.078	0.002	<0.001	0.103	0.004	<0.001	<0.001	0.169	0.018	0,019

Notations: a2, length of segment of second antennomer; a3, length of segment of third antennomer; a4, length of segment of fourth antennomer; pm2, the length of second palpomer of maxillary palpus; pm3l, length of third palpomer of maxillary palpus; pm3w, the width of third palpomer of maxillary palpus; pl1, distance between the two extreme setae on the first palpomer of labial palpus; pl2l, length of second palpomer of labial palpus; pl2w, width of second palpomere of labial palpus; femur, length of femur in front leg; tibia, length of tibia in front leg; elytra, length of elytra. n.m. means that parameters were not measured.

Significant differences were found between males and females for the length of the third and fourth segments of the antenna, the length and width of the third palpomer of the maxillary palpus, the length and width of the second palpomer of labial palpus, and the lengths of the femur and the elytra. The length of the third segment of the antenna was greater in *A. parallelepipedus* and *C. violaceus* males than in females. The fourth segment of the antenna was also longer in *A. parallelepipedus* males than in females. The length of the third palpomer of the maxillary palpus, the width of the third palpomer of the maxillary palpus, and the length of the second palpomer of the labial palpus were greater in *C. violaceus* males than in females. The width of the second palpomer of the labial palpus was greater in *A. parallelepipedus* and *C. violaceus* males than in females. The length of the femur was greater in *A. parallelepipedus* males than in females. The elytra lengths of *C. scheidleri*, *C. violaceus*, and *P. oblongopunctatus* in females were greater than in males (Tables 1–3).

Studying the interaction between urbanization and sex we found significant differences in the following traits: all tested antennomers in *C. scheidleri* and *C. violaceus*, the width of the third palpomer of the maxillary palpus, and the distance between the two extreme setae on the first palpomer of the labial palpus in *C. scheidleri*, the width of the second palpomer of the labial palpus in *A. parallelepipedus*, the length of femur and tibia in *P. oblongopunctatus*, and the length of the elytra in *C. violaceus* and *P. oblongopunctatus*.

Significant asymmetry was found in the lengths and widths of the third palpomer of the right and left maxillary palpus and the widths of the second palpomer of the right and left labial palpus for all tested species along the gradient (Tables 1–3).

## Discussion

Morphometric abnormalities are frequently caused by pollution and/or unfavorable habitat conditions (Saunders et al. 1991, Cachada et al. 2013). We found differences along the urbanization gradient based on the following parameters of the studied traits: antennomers, the maxillary palpus, the labial palpus, and the length of tibia and the elytra. Weller and Ganzhorn (2003) also found morphological changes along the urbanization gradient where the body length of *Carabus nemoralis* declined toward the city center. Maryański et al. (2002) found that metal-contaminated food also had morphometric effects on *Poecilus cupreus* L. under laboratory conditions. They found morphometric effects of lifetime exposure in the case of both zinc and cadmium; these differences were highly significant. In metal-treated individuals, the lengths of the middle femur and front tibia were significantly reduced. Maryański et al. (2002) concluded that individuals exposed to metal-contaminated food concentrated less energy on growth and fat accumulation and more energy on the detoxification process.

Our results suggested that in the cases of the species of Carabini tribus, the antennomers, the maxillary palpus, and the elytra could be useful for assessing the effects of urbanization, whereas in the case of species of Pterostichini tribus, the most useful would be the antennomers and the tibia. Smaller carabid species were more abundant in highly disturbed urban sites; in contrast, large species, which are flightless and therefore have limited dispersal power, are more abundant in a mosaic landscape (Den Boer 1990, Blake et al. 1994, Ribera et al. 2001). Generalist species that occur at all sites and prefer disturbed areas are more resistant to disturbance. Due to their resistance, these species is less affected by urbanization, and the urbanization effects are less traceable by measuring parameters of body parts (Weller and Ganzhorn 2003). Sukhodolskaya (2013) found that the morphometric traits of *Carabus granulatus* specimens were higher in suburban habitats than in natural ones. They also found that forested urban habitats are more heterogeneous than homogenous natural areas in size and food supply. In urbanized areas, the elevated temperature may be an additional component in influencing the development and life cycle of beetles, but in these areas, contamination and fragmentation constitute the major negative pressures on sensitive carabid species. Stone et al. (2001) showed that *P. oblongopunctatus* from chronically metal-polluted sites were less tolerant to additional environmental stressors; the study examined the relationship between metal toxicity and the state of energy reserves and metabolic rates of *P. oblongopunctatus*.

In our study, we found significant differences between male and female individuals based on the parameters of the antennomers, the maxillary palpus, the labial palpus and the length of the tibia, the femur, and the elytra. Under stable environmental conditions, morphological differences between males and females can be explained by their roles in reproduction. For example, the energy allocation of females is concentrated on gamete production and contributes greatly to the explanation of sexual size dimorphism (Nylin and Gotthard 1998, Tammaru et al. 2002). Thiele (1977) and Lagisz (2008) demonstrated that the elytra length of *P. oblongopunctatus* females was significantly larger than in males, which is typical in carabid species. Similarly to these studies, we found that the elytra length of female *C. scheidleri*, *C. violaceus*, and *P. oblongopunctatus* was larger than it was in males. The effects of environmental stress can disrupt the normal energy distribution of the beetles’ body (Cortet et al. 1999). Thus, the different reaction of males and females to external stress is one of the most decisive reasons for their divergent morphological appearance (Badyaev 2002). Sukhodolskaya (2013) demonstrated that urbanization significantly contributes to elytra length variation. In urban habitats, *Carabus aeruginosus* males’ elytra length increased but that of females decreased, and in *C. granulatus* and *Pterostichus niger*, this trait did not change significantly along the urban–rural gradient. In rural habitats, the elytra length increased in *Carabus cancellatus* males and in both sexes in *P. cupreus*. In our study, we found morphological differences between sexes only in one species along the tested gradient. In urban areas, the elytra length increased in *P. oblongopunctatus* males, but decreased in females.

Earlier studies demonstrated that the symmetry and body size of arthropods (woodlice, fruit fly) can be influenced by environmental stress (Peters et al. 2001, Trotta et al. 2005, Vilisics et al. 2005, Godet et al. 2011). The influence of special conditions in urbanized areas on the morphometry of ground beetle species is very variable and contradictory (Braun et al. 2004). Weller and Ganzhorn (2003) tested the fluctuating asymmetry (FA) of carabid species along an urban–rural gradient. In the tested species, including *A. parallelepipedus*, *C. violaceus*, and *P. oblongopunctatus*, the asymmetry of the elytra increased toward the city center and with increasing isolation of the studied areas. In contrast to these studies, Henríquez et al. (2009) demonstrated that the asymmetry of the posterior femur of *Carabus chilensis* individuals did not increase in fragmented habitats. Elek et al. (2014) tested the asymmetry of the lengths of the antennomers, the elytra, the first and second tibiae, the first tarsi and the distance between the end of the femur, and the proximal and distal spines on the first femora of *C. nemoralis*, *Nebria brevicollis*, and *Pterostichus melanarius*. The authors did not find any increase in FA along an urbanization gradient. Maryański et al. (2002) demonstrated that zinc- or cadmium-contaminated food had no effect on symmetry in major and minor axes of the elytra, nor on the length of the front, middle, and hind femur and tibia of *P. cupreus*. In our study, the symmetry of the lengths and widths of the third palpomer of the right and left maxillary palpus and the widths of the second palpomer of the right and left labial palpus changed along the gradient for all species. Differences in the sensitivity between the traits showed that the occurrence of asymmetry also depends on many internal factors. Stress does not affect all tested traits similarly because particular groups of traits and/or organisms may provide stronger asymmetry–stress relationships (Leung and Forbes 1996, Anciães and Marini 2000, Lens et al. 2002). In our study, asymmetry was found only for a few parts of the body. We found that the symmetry of the maxillary palpus and the labial palpus could be useful in estimating anthropogenic effects. The asymmetry may reflect the development of surviving individuals (Floate and Fox 2000); maintaining symmetry may be more important for survival and reproduction than maintaining normal and/or large body size.

### Conclusion

We demonstrated that the effect of urbanization on the body size and on the asymmetry of morphometric characters is a complex process. We found that urbanization influenced the morphology of carabid beetles; urbanization influenced the morphological traits of each species. We also demonstrated that male and female specimen sensitivity to urbanization was different. The species of Carabini tribus and females of the species of Pterostichini tribus responded sensitively to anthropogenic influences. In the case of asymmetry, the variance between traits showed that particular groups of traits and/or organisms may provide stronger asymmetry–stress relationships. We found that the symmetry of the left and right antennomers, maxillary palpus, labial palpus, tibia, and femur is useful in estimating the effect of urbanization. Our results also suggested that the elytra provided the strongest size–stress relationships, and that large species, and females of small species such as *P. oblongopunctatus*, could be useful for assessing the effects of urbanization because they respond sensitively to the anthropogenic effects.
